# The acceleration of glucose accumulation in renal cell carcinoma assessed by FDG PET/CT demonstrated acquisition of resistance to tyrosine kinase inhibitor therapy

**DOI:** 10.1186/s12885-016-3044-0

**Published:** 2017-01-09

**Authors:** Noboru Nakaigawa, Keiichi Kondo, Daiki Ueno, Kazuhiro Namura, Kazuhide Makiyama, Kazuki Kobayashi, Koichi Shioi, Ichiro Ikeda, Takeshi Kishida, Tomohiro Kaneta, Ryogo Minamimoto, Ukihide Tateishi, Tomio Inoue, Masahiro Yao

**Affiliations:** 1 Department of Urology, Yokohama City University Graduate School of Medicine, 3-9 Fukuura Kanazawaku, Yokohama, 236-0004 Japan; 2 Department of Urology, Yokosuka Kyosai Hospital, Yokosuka, Japan; 3 Department of Urology, Yokohama Minami kyosai Hospital, Yokohama, Japan; 4 Department of Urology, Kanagawa Cancer Center, Yokohama, Japan; 5 Department of Radiology, Yokohama City University Graduate School of Medicine, Yokohama, Japan

**Keywords:** Renal cell carcinoma, Tyrosine kinase inhibitor, Resistance acquisition, FDG PET/CT, Standardized uptake value

## Abstract

**Background:**

Tyrosine-kinase inhibitor (TKI) targeting angiogenesis improves the prognosis of patients with metastatic renal cell carcinoma (RCC), but its effect is temporary. In order to understand the mechanism by which RCC acquires resistance to TKI, we investigated the change of glucose accumulation in RCC by FDG PET/CT when they demonstrated progression disease (PD) against TKI.

**Methods:**

We monitored the FDG accumulation in RCC of 38 patients treated with TKI by 162 PET/CT sequentially until they were judged to demonstrate PD. Standardized uptake value (SUV), a simplified index of tissue FDG accumulation rate, was measured, and the sequential changes of max SUVmax (the highest SUV in an individual patient) was analyzed. Additionally, the expression of glucose transporter 1 (GLUT-1) and associated proteins in 786-O cells cultured under hypoxia were analyzed.

**Results:**

The 10 patients with RCC which FDG accumulation was accelerated after beginning of TKI treatment demonstrated PD soon. The other 28 patients with RCC which FDG accumulation was suppressed by TKI showed longer progression-free survival (3.6 months vs 6.5 months, *P* = 0.0026), but this suppression in most cases (96%) was temporary and FDG accumulation was accelerated when tumor demonstrated PD. Interestingly, the FDG accumulation at PD was higher than that before TKI treatment in the half cases. The acceleration of FDG accumulation was suppressed by following treatment by mammalian target of rapamycin (mTOR) inhibitor. Additionally, in vitro assay demonstrated that the expression of GLUT-1 was increased in the RCC cells surviving under hypoxia condition via mTOR pathway.

**Conclusions:**

The acceleration of glucose accumulation dependent on mTOR in RCC assessed by FDG PET/CT demonstrated acquisition of resistance to TKI. FDG PET/CT had potential as an assessment method monitoring not only the initial response but also following status of RCC during TKI treatment.

**Trial registration:**

UMIN0000008141, 11 Jun 2012. This trial was retrospectively registered.

## Background

Renal cell carcinoma (RCC) is the most common type of kidney cancer and accounts for 2–3% of all cancers cases [1, 2]. About 30% patients are diagnosed as metastatic disease and 20–40% of patients with localized disease treated by curative nephrectomy develop metastases [3]. The standard systematic therapies for metastatic RCC had been mainly cytokines including interferon-α and interleukin-2 for a long time, and the outcome was far from satisfactory [4, 5]. With the elucidation of the oncogenic mechanisms of RCC, tyrosine-kinase inhibitors (TKIs) targeting vascular endothelial growth factor (VEGF) receptors such as sunitinib and sorafenib, were developed and the prognosis of patients with metastatic RCC was improved [6–8].

The anti-tumor mechanism of TKs is suppressing the biological activity of RCC by inhibiting angiogenesis. In the phase III studies for these TKI, only 2–31% of patients with tumors showed a partial response (PR) or better as defined by RECIST criteria [6, 7]. Practically, certain RCCs treated with TKIs do not show an obvious reduction in tumor size, but do enter long-term dormancy. Therefore, to evaluate TKI response, it is not enough to merely assess the change in tumor volume by conventional computed tomography (CT) imaging. In this era of molecular targeting therapeutics, a novel index for assessing the biological response of RCC to TKI treatment is desirable.

^18^F-2-fluoro-2-deoxyglucose positron emission tomography/computed tomography (FDG PET/CT) is a functional imaging technique evaluating glucose metabolic status, which can be used as an index of the biological activity of cancer. We have been investigating the usefulness of standardized uptake value (SUV) evaluated by FDG PET/CT, which is a semiquantitative simplified measurement of the tissue FDG accumulation rate, as an index for assessing the biological activity of cancer.

We first found that pretreatment maximum SUVmax (max SUVmax), which was the highest SUV in all RCC lesions in individual patients, could predict the survival of patients with advanced RCC [9, 10]. Subsequently, we reported that patients with RCC whose max SUVmax decreased 20% or more one month after beginning TKI treatment showed long progression-free survival (PFS) [11]. In this study, we investigated the change of max SUVmax when RCC demonstrated progression of disease (PD) against TKI treatments defined by RECIST criteria, in order to understand the mechanism how RCC acquires resistance to TKI treatment.

## Methods

### Patients and interventions

The patients planning to undergo TKI therapy for pathologically proven metastatic RCC at Yokohama City University Hospital (Yokohama, Japan) and affiliated institutions between June 2008 and July 2013 were enrolled in this prospective study. Patients with uncontrolled diabetes mellitus (FBS > 150 mg/dl), other known malignancies, or who were treated systemically within the last four weeks were excluded.

**Fig. 1:**
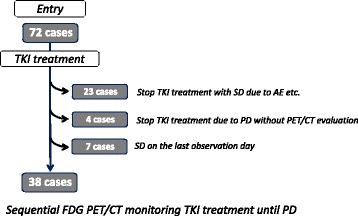
Patient disposition of this clinical trial monitoring renal cell carcinoma by FDG PET/CT during TKI treatment until tumor demonstrated progression disease

Seventy-two patients were initially enrolled in this study. Of these, 34 were eliminated: 23 patients showing stable disease (SD) stopped TKI treatment due to an adverse event, change of treatment strategy, at their request, general prostration, or other disease; 4 patients showed SD on the last observation day; and 7 patients were diagnosed as PD by unplanned CT scan due to some symptom, and were not evaluated by PET/CT. Thus, participants in the study were 38 patients who continued treatment with TKI and were evaluated by FDG PET/CT sequentially until their RCC showed PD as defined by the Response Evaluation Criteria in Solid Tumors (RECIST) version 1.1 criteria [12] (Fig. 1).

### Imaging

FDG PET/CT was performed before TKI treatment, one month after beginning TKI treatment, and then every three months. Occasionally, the timing of FDG PET/CT assessment was modified for clinical or social reasons. Patients fasted for at least 6 h prior to intravenous injection of ^18^F FDG. PET/CT images were obtained using a single PET/CT system (Aquiduo 16; Toshiba Medical Systems, Tokyo, Japan) at the Yokohaha City University Hospital. The details about the condition of PET/CT examination and methods how to determine Standardized uptake value (SUV) were described in our previous report [10]. The highest SUV in an individual RCC lesion and the highest SUV in all RCC tumors of an individual patient were defined as SUVmax and max SUVmax, respectively. Tumor size responses were evaluated by the RECIST version 1.1 [12]. Two experienced physicians, who were blinded to the clinical data examined each image.

### In vitro assay

786-O cells were purchased from American Type Culture Collection (Manassas, VA, USA). These cell lines were maintained in DMEM containing 10% FCS at 37 °C under 21% O_2_ concentration before analysis under specific conditions. The 786-O cells were cultured under either 21% O_2_ concentration or 5% O_2_ concentration with or without 20nM of rapamycin, purchased from Sigma (St. Louis, MO, USA) for 2 days before harvesting. Cultured cells were harvested under appropriate conditions. Cells were washed twice with ice cold phosphate-buffered saline (PBS), lysed in ice cold buffer consisting of 20 mM Tris (pH 8.0), 137 mM NaCl, 10% glycerol, 0.1% SDS, 0.5% Nonidet P-40, 100 mM sodium fluoride, 200 mM sodium orthovandate, 1 mM EGTA, 2 mM phenylmethylsulfonyl fluoride, 1 μg/ml leupeptin, and 3 μg/ml aprotinin, then centrifuged (30 min, 4 °C, 14,500Xg). Following quantitation, 30 μg of lysates was subjected to SDS-PAGE on appropriate gel, and electorotransferred to Immobilon-P (Millipore, Bedford, MA, USA). After blocking the membrane with 5% albumin, Western blotting was performed and detected with an ECL detection system (Amersham Int., UK). Anti-human HIF-2α antibodies were purchased from Novus Biological (Littleton, CO, USA). Anti-GLUT1 antibodies were purchased from Santa Cruz Biotechnology (Santa Cruz, CA, USA). Anti-Cyclin D1 antibodies were purchased from PharMingen (San Diego, CA, USA). Anti-actin antibodies were purchased from Sigma (St. Louis, MO, USA).

### Statistical analysis

PFS time was calculated from the date TKI treatment began to the date on which RCC demonstrated PD, as defined by RECIST criteria version 1.1 [12]. PFS was estimated by the Kaplan-Meier method, and compared using the log-rank test. The impact of SUVmax change on the diameter change of tumors was analyzed by two-sided Mann-Whitney’s *U* test. All statistical analyses were carried out with SPSS software (SPSS, Inc., Chicago, IL).

## Results

### Patient characteristics

A total of 38 patients (32 males and 6 females) were analyzed in this study (Table 1, Fig. 1). Median age was 65 years (range 32–82). There were 25 patients with recurrent disease and 13 with stage IV disease. Of the 25 patients with recurrent disease, the 12 patients had recurrence within 5 years after nephrectomy, and the other 13 patients did after more or 5 years. Pathological examination revealed 32 cases of clear cell carcinoma, 5 of papillary, and 1 of unclassified renal cell carcinoma. The number of metastases in individual patients was 1 to 10 (median 3.0). Thirty-two patients had undergone nephrectomy, while 23 had not undergone previous systematic therapies. The diameter of primary carcinoma was 2.7 to 11.8 cm (median 6.0). Of 24 primary carcinoma which detailed pathological findings could confirmed, 13 (54%) included tumor necrosis. Ten patients had undergone cytokine therapies, 2 cytokine and sorafenib, 1 sorafenib, 1 chemotherapy, and 1 temsirolimus. These previous treatments had ended more than 4 weeks before the pretreatment evaluation by FDG PET/CT.

**Table 1:** Characteristic of patients

Age	32–82 y.o. (median 65 y.o.)	
Gender	Male 32	Female 6
Histology	Clear cell	32
	Papillary	5
	Unclassified	1
Nephrectomy	Yes	32
	No	6
MSKCC classification		
	Favorable	9
	Intermediate	25
	Poor	4
Prior systematic treatment		
	No	23
	Cytokine	10
	Cytokine andsorafenib	2
	Sunitnib	1
	Chemotherapy	1
	Temsirolimus	1

For this study, 20 patients were treated with sorafenib, and 18 with sunitinib. The median PFS of all cases was 5.4 months (range, 0.9 to 32.3).

The patients were evaluated by FDG PET/CT 2 to 13 times (median 4 times). The total number of FDG PET/CT evaluation was 162.

### Change of FDG accumulation during TKI treatment

**Fig. 2:**
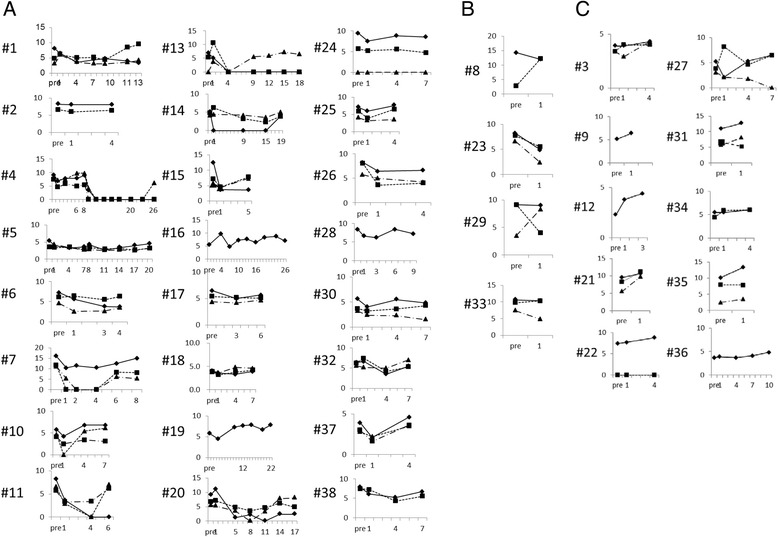
The sequential change of SUVmax during TKI treatment in 38 cases: **a** 24 cases showed decrease of max SUVmax and continued TKI treatment. **b** Four cases showed PD on the first evaluation after the start of TKI treatment despite the decrease in max SUVmax. **c** 10 cases showed increase of max SUVmax. The *vertical* axis shows SUVmax of individual RCC lesions. In cases with more than three RCC lesions, we used the SUVmax in the three lesions where SUVmax was highest in the pretreatment evaluation. The *horizontal* axis represents time of FDG PET/CT evaluation. “Pre” means pretreatment evaluation; numbers refer to number of months after TKI treatment started

We investigated the change of FDG accumulation focusing on max SUVmax, which was the highest SUV in all RCC lesions in individual patients (Table 2). The pretreatment max SUVmax of all patients ranged from 2.3 to 16.1 (median 6.8). In 28 cases (74%), max SUVmax decreased after the start of TKI treatment (Fig. 2a and b); in 10 cases (26%), it increased (Fig. 2c). The median PFS of the 28 cases with decreasing max SUVmax was 6.5 months (95% CI 5.8–7.2 months), while that of the 10 cases with increasing max SUVmax was 3.6 months (95% CI 1.5–5.7 months). This difference was statistically significant (log-rank *P* = 0.0026).

**Table 2:** Patients and sequential change of max SUVmax

Pt. #	Age	Treatment	MSKCC classification	max SUVmax before treatment	max SUVmax at nadir	max SUVmax at PD	PFS (month)	Number of PET/CT evaluation
1	70′s	sorefanib	favorable	8.1	4.8	9.5	15.0	7
2	60′s	sorefanib	intermediate	8.2	8.0	8.0	3.2	3
3	50′s	sunitnib	intermediate	3.9	not decrease	4.0	4.1	3
4	50′s	sunitnib	intermediate	9.1	0.0	6.2	26.1	13
5	70′s	sorefanib	intermediate	5.3	2.7	4.6	21.0	9
6	60′s	sorefanib	intermediate	7.2	5.5	6.3	5.5	4
7	30′s	sunitnib	favorable	16.1	10.4	15.0	7.8	6
8	60′s	sunitnib	poor	14.3	12.2	12.2	0.9	2
9	80′s	sunitnib	intermediate	5.1	not decrease	6.4	2.2	2
10	50′s	sunitnib	intermediate	5.8	4.2	6.8	7.4	4
11	60′s	sunitnib	intermediate	8.2	3.4	7.0	6.1	4
12	70′s	sorefanib	intermediate	2.3	not decrease	4.4	3.6	3
13	80′s	sorefanib	intermediate	6.9	0.0	7.6	18.5	7
14	50′s	sunitnib	favorable	5.2	3.6	5.1	19.4	5
15	50′s	sunitnib	favorable	12.5	4.5	7.8	4.8	3
16	60′s	sorefanib	favorable	5.2	4.8	7.1	26.2	9
17	70′s	sorefanib	intermediate	6.5	5.2	5.7	6.5	3
18	60′s	sorefanib	favorable	4.1	3.6	4.6	6.9	4
19	50′s	sorefanib	favorable	5.8	4.5	7.8	22.3	7
20	70′s	sunitnib	intermediate	9.1	3.6	8.1	17.4	7
21	50′s	sorefanib	poor	9.5	not decrease	12.0	1.4	2
22	60′s	sorefanib	intermediate	7.4	not decrease	8.8	3.6	3
23	50′s	sunitnib	intermediate	8.2	5.5	5.5	0.9	2
24	70′s	sorefanib	intermediate	9.4	7.5	8.5	6.2	4
25	70′s	sorefanib	intermediate	7.0	5.9	7.6	4.2	3
26	60′s	sunitnib	poor	8.2	6.4	6.6	3.4	3
27	60′s	sorefanib	intermediate	5.2	not decrease	6.5	7.0	4
28	70′s	sunitnib	intermediate	8.4	6.2	7.2	10.7	5
29	50′s	sunitnib	poor	9.1	9.0	9.0	0.9	2
30	60′s	sorefanib	favorable	5.6	4.0	4.8	6.9	4
31	50′s	sunitnib	intermediate	11.0	not decrease	12.8	1.5	2
32	70′s	sunitnib	intermediate	6.4	4.2	7.0	6.5	4
33	60′s	sunitnib	intermediate	10.6	10.3	10.3	1.0	2
34	60′s	sorefanib	intermediate	5.5	not decrease	6.0	4.7	3
35	60′s	sorefanib	intermediate	10.1	not decrease	13.3	0.9	2
36	60′s	sunitnib	intermediate	3.7	not decrease	4.8	9.9	5
37	70′s	sorefanib	favorable	3.9	2.2	4.6	6.5	3
38	60′s	sunitnib	intermediate	7.9	5.2	6.7	2.9	4

**Fig. 3:**
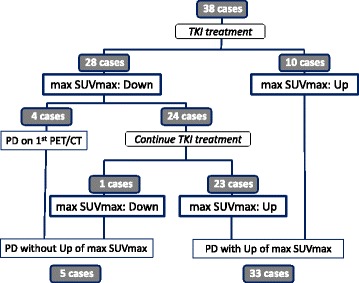
The sequential change of max SUVmax during TKI treatment in all 38 cases was summarized

In the 28 cases with decreasing max SUVmax, the median date when SUVmax reached its nadir was 61 days. In 14 cases (50%), max SUVmax showed a nadir at the evaluation 1 month after the start of TKI. The median decreasing ratio of SUVmax at the nadir compared with that before treatment was 28% (1–100). Four cases showed PD on the first evaluation after the start of TKI treatment despite the decrease in max SUVmax (Fig. 2b). The other 24 cases continued TKI treatment, and the max SUVmax increased after nadir in 23 cases (96%) (Fig. 2a and Fig. 3). Max SUVmax in 12 cases (52%) increased before cancer showed PD, and that in other 11 cases increased on the same examination when cancer showed PD. The time lag between max SUVmax increase and PD was 0–476 days (median 47 days). In 33 of the 38 cases (87%), max SUVmax increased until RCC showed PD (Fig. 3). A comparison of max SUVmax at PD and before TKI treatment showed that the SUVmax at PD was higher in 19 of the 38 cases (50%).

**Fig. 4:**
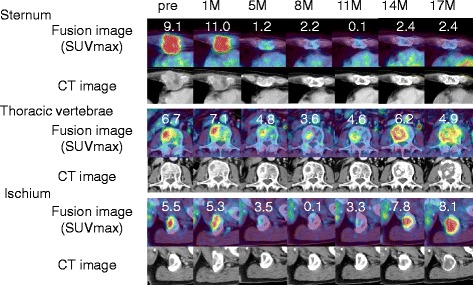
The imaging of a practice case: 70’s-year-old patient with sternum, thoracic vertebrae, and ischium bone metastases (#20). The SUVmax of all bone metastases decreased temporally and increased before tumor was judged as PD. Fusion images were upper lanes. The number means SUVmax of each lesion. CT images were lower lanes. “Pre” refers to pretreatment evaluation; “M” refer to month after TKI treatment started

Next, we investigated the change of the highest SUV in individual RCC lesions (SUVmax) in the 32 patients who had two or more RCC lesions in order to know whether multiple lesions in one patient showed similar or independent responses to TKI treatment. In 13 of 32 cases (41%), the SUVmax of one lesion increased more than 10% while that of the other decreased more than 10% compared with the SUVmax in the previous evaluation.

To reveal the association between FDG accumulation and anatomical change in each lesion, we focused on the 123 lesions which SUVmax changed more than 20% compared with previous examination. Of the 60 lesions which SUVmax increase more than 20%, 34 lesions (57%) demonstrated the enlargement of diameters simultaneously, 14 (23%) did not change and other12 (20%) reduced. Otherwise, of 63 lesions which SUVmax decrease, 39 lesions (62%) demonstrated the reduction of diameters simultaneously, 13 (21%) did not change and other 11 lesions (17%) grew larger. There was significant difference between these two groups (*P* < 0.001).

**Fig. 5:**
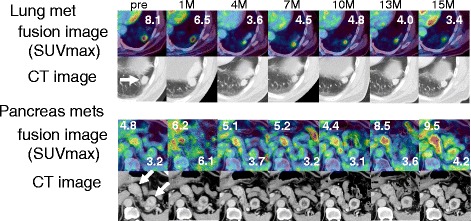
The imaging of a practice case: 70’s-year-old patient with lung and pancreas (head and tail) metastases treated by sorafenib (#1). The SUVmax of lung metastasis had suppressed during treatment, but the SUVmax of pancreas head metastasis increased before tumor was judged as PD. Fusion images were *upper lanes*. The number means SUVmax of each lesion. CT images were *lower lanes*. “Pre” refers to pretreatment evaluation; “M” refer to number of month after TKI treatment started

The imaging of a practice case was shown in Fig. 4 and 5.

### The acceleration of glucose accumulation during TKI treatment was suppressed by mTOR inhibitor

**Fig. 6:**
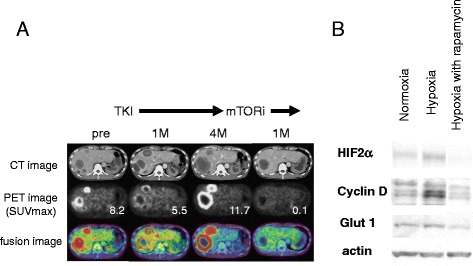
Accelerated FDG accumulation during TKI treatment can be dependent on mTOR activity. **a** 50’s-year-old patient with liver metastases (#23). The FDG accumulation of liver metastasis that accelerated during 4 months of sunitinib treatment was suppressed by 1 month of everolimus treatment. **b** The expression of GLUT-1, Cyclin D, and HIF-2 α was accelerated in the 786-O cells that survived under a hypoxic condition. The overexpression of these proteins induced by hypoxia was suppressed by 20nM rapamycin

Of 33 cases with their max SUVmax increasing during TKI treatment, 6 cases was sequentially treated by everolimus, an oral mTOR inhibitor. Interestingly, the max SUVmax of the all 6 cases was decreased by everolimus, oral mammalian target of rapamycin (mTOR) inhibitor as shown in Fig. 6a. In order to disclose the mechanism how FDG accumulation was accelerated in RCC when oxygen supply was suppressed by TKI inhibiting angiogenesis and the association between this mechanism and mTOR, the expression of protein associated with oncogenesis in 786-O cells, human clear cell RCC cultured under hypoxia was investigated. The expression of glucose transporter-1 (GLUT-1), Cyclin D, and hypoxia-inducible factor 2α (HIF-2 α) was accelerated in the 786-O cells that survived under hypoxic conditions. Additionally, the overexpression of these proteins induced by hypoxia was suppressed when 786-O cells were cultured under the same hypoxic conditions but with rapamycin, a classical mTOR inhibitor (Fig. 6b). These in vitro results suggested that mTOR acitivtiy could be involved in the increase of FDG accumulation in RCC induced during TKI treatment.

## Discussion

We previously reported that patients with advanced RCC in which max SUVmax was decreased 1 month after beginning TKI treatment had long PFS [11]. Several other investigators have reported the usefulness of FDG PET/CT evaluation for assessing the response to TKI treatment [13–15]. These results suggested that FDG accumulation could be an index of the biological response to TKI of advanced RCC. These previous reports, however, focused on the early response to TKI. In our study, we followed patients treated with TKI by FDG PET/CT until RCC showed PD defined by RECIST criteria, to clarify the association between sequential change of glucose accumulation and acquisition of resistance to TKI treatment. This is the first report to reveal the sequential change of FDG accumulation from the beginning to the end of TKI treatment.

We first focused on the response to TKI treatment, and found that the max SUVmax of RCC decreased in 28 of 38 patients (74%). The PFS of these patients was longer than that of the 10 RCC patients whose max SUVmax did not decrease. These results confirmed a previous report that FDG PET/CT was useful for evaluating the early biological response to TKI treatment.

Next, we focused on the 24 patients who continued TKI treatment after their max SUVmax showed nadir. The max SUVmax increased in 23 of 24 patients (96%) when RCC was judged to be PD. Adding the 10 cases with increasing max SUVmax after the start of TKI, the max SUVmax in 33 of our 38 total cases (87%) increased when the RCC was judged as PD. Additionally, the max SUVmax at PD was higher than that before treatment began in 19 patients (50%). Additionally, this acceleration was suppressed by the following treatment by everolimus, oral mTOR inhibitor. These data suggested that the acceleration of glucose uptake associated with mTOR pathway played an important role in acquisition of RCC resistance to TKI.

To know the association of the acceleration of glucose uptake during TKI treatment and mTOR, we observed the expression of protein associated with glucose accumulation in human RCC cells under hypoxic conditions, mimicking the condition of RCC tissue suppressed angiogenesis by TKI treatment. In the 786-O cells, overexpression of GLUT-1 that facilitated the transport of glucose over a plasma membrane was induced under hypoxic conditions. It is well known that the accumulation of HIF induced by alteration of the Von Hippel-Lindau (VHL) gene causes clear cell RCC, and HIF regulates the expression of various genes involved in energy metabolism, angiogenesis, cell proliferation, cell mortality and other biological processes including GLUT-1 and cyclin D [16, 17]. Interestingly, the overexpression of not only GLUT-1 but HIF and cyclin D was overexpressed under hypoxia condition and these overexpression was suppressed by rapamycin, classical mTOR inhibitor. These results suggested that the overexpression of various protein necessary for adapting to hypoxia condition, including GLUT-1 was dependent on mTOR pathway. Recently, three deferent basic research groups reported the novel resistance mechanism of cancer to anti-angiogenic therapy [18–20]. They revealed that the cancer cells adjusted their glucose metabolic status by mTOR pathway regulating the expression of molecule associated with glucose and lactate uptake and acquired the resistance to hypoxia condition induced by anti-angiogenic therapy. Jiménez-Valerio G et al. demonstrated that overexpression of GLUT-1 induced by hypoxia condition acted important roles for resistance to anti-angiogenic therapy in RCC by xenograft model and in vitro assay [18]. Allen E et al. [19] and Pisarsky L [20] reported the similar mechanism in pancreatic neuroendocrine tumors and breast cancer, respectively. Our in vitro data matched these reports, and our clinical results that the acceleration of FDG accumulation demonstrated acquisition of resistance to anti-angiogenic therapy suggested that this novel resistance mechanism identified by basic research acted practically in human cancer treated by angiogenesis inhibitor. It was of course that this mechanism could not explain all of resistance mechanism of RCC to TKI treatment. Various resistance mechanisms of cancer to anti-angiogenic therapy were previously reported from many investigators [21].

Finally, we examined the difference of biological response among multiple RCC lesions in individual patients. We analyzed the sequential change (increase or decrease) of SUVmax in the 2 RCC lesions in 4 patients, and that in the 3 lesions showing the highest SUVmax among all lesions before treatment in 28 patients who had 3 or more RCC lesions. Interestingly, all lesions showed the same pattern, whether it was an increase or decrease (parallel pattern), in only 9 of the 32 cases (28%). In 13 of 32 cases (41%), the SUVmax of one lesion increased more than 10%, while that of the other lesion decreased more than 10%, compared with the SUVmax in the earlier evaluation. Additionally, the lesion that showed max SUVmax changed during TKI treatment in 14 of 32 patients (44%). These results implied that the biological responses of individual lesions were independent in most patients with multiple RCC metastases. This difference could be caused by the heterogeneity of RCC in individual patients, which Gerlinger et al. revealed by genetic analysis [22]. The heterogeneous response suggested that systemic evaluations, for example, assessment using blood biomarker, are insufficient for evaluating the response of RCC, and that imaging biomarker evaluating the response of individual RCC lesions like FDG PET/CT are necessary.

To the best of our knowledge, this is the first report to evaluate the usefulness of monitoring RCC response by FDG PET/CT sequentially during TKI therapy, focusing on the acquisition of resistance to treatment. Although the number of patients analyzed in this study was limited, we revealed that sequential monitoring by FDG PET/CT has potential as an imaging biomarker to allow more effective TKI use. As TKIs can cause various adverse events and be an economic burden on patients, such knowledge could be quite useful. Further study targeting a large number of patients is necessary.

## Conclusions

These data indicate that FDG PET/CT has potential as an assessment method for evaluating the resistance acquisition of RCC to TKI treatment. The increase of glucose accumulation dependent on mTOR can be one of the mechanisms by which RCC acquires resistance to TKI treatment.

## Abbreviations

FDG PET/CT: ^18^F-2-fluoro-2-deoxyglucose positron emission tomography/computed tomography
GLUT-1: Glucose transporter 1
HIF-2 α: Hypoxia-inducible factor 2α
max SUVmax: Maximum SUVmax
mTOR: Mammalian target of rapamycin
PD: Progression disease
PFS: Progression-free survival
PR: Partial response
RCC: Renal cell carcinoma
RECIST: Response evaluation criteria in solid tumors
SD: Stable disease
SUV: Standardized uptake value
TKI: Tyrosine-kinase inhibitor
VEGF: Vascular endothelial growth factor
VHL: Von Hippel-Lindau
VOI: Volume of interest
